# Differential Impact of Stress Reduction Programs upon Ambulatory Blood Pressure among African American Adolescents: Influences of Endothelin-1 Gene and Chronic Stress Exposure

**DOI:** 10.1155/2012/510291

**Published:** 2011-11-24

**Authors:** Mathew J. Gregoski, Vernon A. Barnes, Martha S. Tingen, Yanbin Dong, Haidong Zhu, Frank A. Treiber

**Affiliations:** ^1^Colleges of Nursing and Medicine, Medical University of South Carolina, Charleston, SC 29425, USA; ^2^Georgia Institute for Prevention of Human Diseases and Accidents, Department of Pediatrics, Georgia Health Sciences University, Augusta, GA 30912, USA

## Abstract

Stress-activated gene × environment interactions may contribute to individual variability in blood pressure reductions from behavioral interventions. We investigated effects of endothelin-1 (ET-1) LYS198ASN SNP and discriminatory stress exposure upon impact of 12-week behavioral interventions upon ambulatory BP (ABP) among 162 prehypertensive African American adolescents. Following genotyping, completion of questionnaire battery, and 24-hour ABP monitoring, participants were randomized to health education control (HEC), life skills training (LST), or breathing awareness meditation (BAM). Postintervention ABP was obtained. Significant three-way interactions on ABP changes indicated that among ET-1 SNP carriers, the only group to show reductions was BAM from low chronic stress environments. Among ET-1 SNP noncarriers, under low chronic stress exposure, all approaches worked, especially BAM. Among high stress exposure noncarriers, only BAM resulted in reductions. If these preliminary findings are replicated via ancillary analyses of archival databases and then via efficacy trials, selection of behavioral prescriptions for prehypertensives will be edging closer to being guided by individual's underlying genetic and environmental factors incorporating the healthcare model of personalized preventive medicine.

## 1. Introduction

Essential hypertension (EH) is a major risk factor for cardiovascular disease (CVD), and EH incidence among youth is increasing [1]. African Americans (AAs) experience a higher prevalence, earlier onset, and greater severity of EH-related complications than other ethnic groups [2]. From late childhood onward, AAs display increased levels of resting and ambulatory blood pressure (ABP) compared to other ethnic groups [3–5]. BP levels are monotonically associated with future CVD morbidity and mortality [6]. Stage I prehypertensive adults (i.e., SBP/DBP 121–129/81–84 mmHg) have a 40% increased risk and adults with stage II prehypertension (i.e., SBP/DBP 130–139/85–89 mmHg) are twice as likely to develop CVD compared to those with optimal BP (<120/<80 mmHg) [6–8]. BP percentile ranking tracks from late childhood into adulthood [9–11] placing AA adolescents with BP between the 50th and 95th percentiles for age and sex at an increased risk of future EH and CVD development [9].

EH, like other multifactorial chronic diseases, results from a complex interplay between an individual's genetic underpinnings, lifestyle behaviors, psychosocial factors, and exposures to various environmental toxins. Over time, this dynamic interplay eventuates in adverse structural and functional changes in biological organ systems culminating in disease manifestation [12, 13]. Among the myriad of environmental toxins, psychosocial stress such as repeated exposures to unfair treatment and discrimination associated with socioeconomic status (SES) inequality and race have been implicated as contributing to EH, especially among AAs [14, 15]. 

Few pediatric studies have addressed impact of unfair treatment and discrimination upon BP. Clark and Gochett [14] found perceived racism to be positively associated with increased resting SBP among AA youth who reported a strong intolerance to racist attitudes. Matthews and colleagues observed unfair treatment to be associated with increased daytime ABP and night/day ABP ratios in adolescents [16], especially among AA adolescents living in lower SES neighborhoods [17]. 

AAs' BP control abnormalities are frequently associated with increased vasoconstrictive tone [18–20]. Studies involving normotensive youth and young adults have shown that higher levels of resting BP and exaggerated BP responses to physical and behavioral stressors between AAs and European Americans (EAs) are often due to higher levels and/or greater increases in vasoconstrictive tone [21–24]. Associations between psychosocial stress-related factors and autonomic nervous system (ANS) dysregulation indicate that excessive endothelial activation also plays a contributory role [25]. The endothelial and vascular smooth muscle cells produce endothelin-1 (ET-1), a potent vasoconstrictor, and endothelium-derived relaxing factor (EDRF; a potent vasodilator). Imbalances between circulating concentrations, and/or receptor sensitivity may lead to exacerbations of vasoconstrictive mediated BP control compounding the contributions of ANS dysregulation. Among hypertensive adults and normotensive adolescents and adults, AAs have exhibited higher plasma ET-1 levels compared to EAs [23, 26–28]. Among normotensive adolescents and young adults, AAs have shown greater behavioral stress induced plasma ET-1 increases compared to EAs [23, 26]. A recent study by Cooper et al. found that among AA adults, greater self-reported discrimination exposure was associated with higher ET-1 levels, regardless of SES [29].

The ET-1 gene is localized on chromosome 6, spans 5.5 kb, and contains 5 exons and 4 introns. It has been identified as a candidate gene for EH and CVD [30]. A G-to-T transversion predicting a Lysine-Asparagine change at amino acid 198 (Lys198Asn) single-nucleotide polymorphism (SNP) has been associated with increased BP levels from adolescence to middle age in Japanese EAs and AAs [31–33]. The Lys198Asn SNP has also been associated with exaggerated BP reactivity to laboratory stressors, particularly within the context of background stress-related factors. For example, in a previous study, T allele carriers from lower SES backgrounds exhibited the greatest BP increases to a video game challenge compared to all other subgroups [26]. Rabineau et al. [34] found vasoconstrictive reactivity to behavioral stress was the highest among T-allele carriers with poor anger management skills. These latter sets of findings along with other recent studies [35–37] lend support to the gene × environment model of stress-induced EH [38, 39]. That is, individuals with genetic susceptibility for EH, who are exposed to frequent environmental stress and/or other stress-related potentiating factors (e.g., ineffective coping skills), will be most likely to exhibit the greatest BP stress reactivity and to eventually develop EH and CVD.

Behavioral stress reduction interventions (e.g., meditation, cognitive behavioral coping skills, etc.) implemented to improve BP control and other CVD risk factors have primarily involved adults and quality of research designs and results have been mixed [40–42]. Rainforth et al. [42] reviewed 107 stress reduction BP control studies and conducted a meta-analysis involving 20 studies that were classified as well-designed randomized control trials (RCTs). All but two of the studies involved prehypertensive and hypertensive adults. Collectively, Transcendental Meditation (TM) was the only treatment found to significantly reduce resting and/or 24 hour BP.

 Far fewer RCTs have been conducted involving youth, but findings are promising. Black et al. [43] reviewed 16 pediatric sitting-meditation RCTs, including breathing awareness meditation (BAM). Median effect sizes ranged from 0.16 to 0.29 for physiologic outcomes including resting and ambulatory BP, heart rate, and total peripheral resistance [43]. In a recent study, BAM showed significantly greater reductions in ambulatory SBP and sodium excretion compared to cognitive behavioral skills training (LST) and health education control (HEC) among a group of AA prehypertensive teenagers [44]. Increased SNS activation and increased endothelial system activity (i.e., increased ET-1 levels) have both been shown to increase sodium appetite [45, 46]. The reduced sodium excretion may be indicative of reductions in sodium appetite as a result of improvements in ANS regulation and/or ET-1 activity.

The above review indicates that among stress reduction RCTs, meditation is consistently associated with significant BP reductions. However, even among the meditation RCTs showing significant BP reductions, noticeable inter-individual differences have been observed both across and within studies [41–44, 47]. For example, in Rainforth et al.'s [42] meta-analysis, the 95% confidence interval for resting SBP change from 6 TM studies was (−2.3 to −7.6 mmHg), with net changes between TM and health education ranging from −1.1 to −10.7 mmHg. Recently, in a group of college students, Nidich et al. [48] found TM to provide an average change of −2.0 mmHg for resting SBP. A subgroup identified as high-risk for EH (i.e., family history of EH) showed a reduction of −5.0 mmHg.

The variability within and across RCTs using meditation interventions with comparable study samples and adherence rates may partially be due to combined influences of heterogeneity in genetic susceptibility for physiological responsivity to stress and propensity for exposure to stressful events. In genetics, penetrance represents the percentage of cases carrying a gene or allele among those displaying the phenotype of interest; expressivity represents variations or magnitude in a phenotype expression. Phenotypes can vary in penetrance and expressivity by a number of factors including exposure to environmental factors, allelic variation, and complex gene by environment interactions [49]. A growing literature is indicating that higher genetic penetrance and/or expressivity may adversely impact the degree of benefit obtained from behavioral as well as pharmacologic programs aimed at improving relevant phenotypes. For example, among smokers who participated in cognitive behavioral interventions plus pharmacologic smoking cessation therapies, the carriers of nicotine metabolizing genetic variants, particularly those from high tobacco smoke exposure-laden environments, exhibited lower cessation rates and earlier relapse compared to noncarriers [50]. Similarly, ancillary analyses of the highly successful Diabetes Prevention Program which involved at-risk adults from obese laden environments receiving placebo, metformin, and lifestyle interventions revealed carriers of the TCF7L2 genetic variant associated with diabetes, had significantly higher incidence of diabetes acquisition during the study than noncarriers irrespective of the treatment they received [51]. 

The purpose of this preliminary study was to evaluate the potential modulating influences of genetic variability in the ET-1 SNP and differential discrimination-based stress exposure upon changes in ambulatory BP after 12-week exposure to BAM, LST, and HEC among prehypertensive AA adolescents. We hypothesize that ET-1 carriers will be less likely to respond to any stress reduction treatment given the purported propensity of a greater genetic predisposition to ANS/ET-1 imbalances related to BP control. Following, we expect ET-1 carriers who report high levels of discrimination will have the most difficulty reducing ambulatory BP compared to other subgroups due to the combination of having higher genetic penetrance for an ANS/ET1 imbalance related to BP control, combined with increased likelihood of expressivity of the ANS/ET1 imbalance as a result of high levels of chronic stress exposure. Among the treatment groups, we hypothesized that BAM would have greater beneficial impact upon ambulatory BP reduction compared to LST and HEC. 

## 2. Methods

### 2.1. Subjects

**Figure 1:**
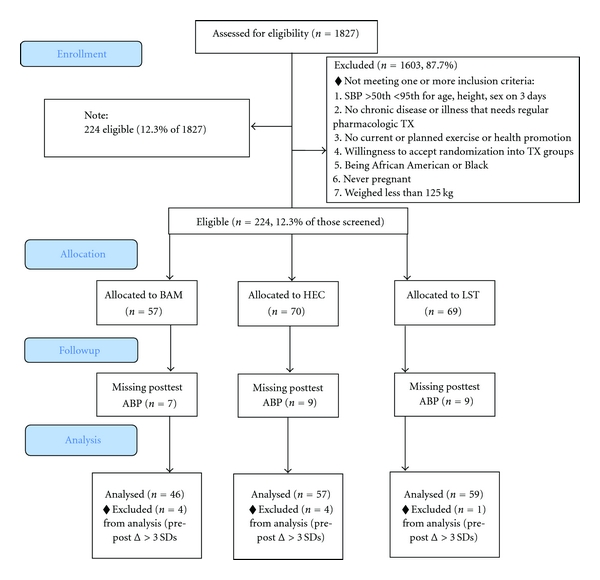
Consort diagram of participant distribution.

As shown in Figure 1, a total of 1827 students who would be participating in a semester-long ninth grade health education class were screened over a five-year period to determine eligibility for participation in the study. Eligibility criteria included having (1) resting SBP between 50th and 95th percentile for age, height and sex [52] on three consecutive occasions at school, (2) no history of congenital heart defect, diabetes, sickle cell anemia, asthma, or any chronic illness or health problem that requires regular pharmacological treatment, (3) no current or planned engagement in a formal exercise, health promotion, or organized sports program outside of regular school physical education courses, (4) willingness to accept randomization by school into treatment groups, (5) being “African American” or “Black”, based on parental report, (6) never pregnant at any point in the study, and (7) weighing less than 125 kg. The Institutional Review Boards of the Georgia Health Sciences University and the Medical University of South Carolina approved the study.

 From the 224 eligible participants, genotyping was not conducted on 30 students. Thirty-two were omitted due to either missing postevaluation ambulatory BP (*n* = 23), or having extreme changes (*n* = 9) in postintervention 24-hour SBP (≥3 SDs compared to the entire sample; 15 mmHg in either direction). 

The distribution of the remaining 162 subjects by treatment group was: BAM (*n* = 46, 16 males), LST (*n* = 59, 24 males), and HEC (*n* = 57, 20 males). There was no differential loss of subjects by treatment group (*X* ^2^ = 3.65, *df* = 2, *P* = .72) and no significant differences between omitted subjects (*n* = 62) and the remaining 162 on anthropometric variables and ambulatory BP levels at baseline (all  *P*'s >.10).

### 2.2. Procedures

#### 2.2.1. BP Screening

Three consecutive days of school screenings were conducted. Height was measured by stadiometer and weight by a Detecto CN20 scale (Cardinal Scale Manufacturing Co., Webb City, Mo, USA). Seated SBP was recorded using Dinamap 1846SX monitors (Critikon, Inc., Tampa, Fla, USA) at minutes 5, 7, and 9 of a 10-minute rest period. The first measurement each day was discarded and the other two measurements were averaged*.*

#### 2.2.2. Genotyping

Genomic DNA was extracted from buccal cells using QiaAmp DNA Blood Mini Kits (Qiagen). Extracted DNA was stored at −80°C until analyzed. The ET-1 Lyn198Asn genotype was detected by polymerase chain reaction (PCR) followed by direct sequence analysis [31].

#### 2.2.3. Ambulatory Blood Pressure Evaluation

Before and following the intervention, ambulatory SBP and DBP were recorded for 24 hours. Measurements were recorded every 30 minutes during school, every 20 minutes during self-reported after school waking hours, and every 30 minutes during self-reported sleep hours using Spacelabs 90207 monitors (SpaceLabs, Inc., Issaquah, Wash, USA). This monitor has been previously validated, and ambulatory BP has been found to be a better predictor of EH than casual BP [53]. Acceptability of ambulatory readings was based on previously established criteria including pulse pressure >20 mmHg, DBP ≥ 45 mmHg but <100 mmHg, SBP > 70 mmHg but <180 mmHg, HR > 39 bpm but <180 bpm [47, 54, 55]. Hourly averages were obtained by averaging all readings for each clock hour across: daytime (8 a.m. to 10 p.m.), nighttime (12 a.m. to 6 a.m.), and 24-hour periods. As in previous studies, to be included in analyses, hourly averages for SBP and DBP required a minimal of 50% of total possible evaluations for the respective time period [47, 54, 55]. The percentage of ambulatory evaluations across groups for the pre/postinterventions were 78%|80%. The percentage of ambulatory evaluations by group for the pre/postinterventions were similar: BAM 78%|81%, LST 78%|79%, and HEC 79%|80%.

#### 2.2.4. Discrimination Assessment

The 9-item everyday discrimination scale (EDS) was used to assess exposure to discrimination [56, 57]. Frequency of encounters was assessed using a 6-point response format (almost every day, at least once a week, a few times a month, a few times a year, less than once a year, and never). The EDS was administered via paper and pencil during pre- and postintervention evaluations as part of a battery of psychosocial questionnaires. The EDS has good internal consistency *α* = .88 [58], and unidimensional factor structure [56]. Cronbach's *α* was.84 in the current sample of preintervention data. To investigate influence of everyday discrimination, a median split was conducted on preintervention EDS scores creating low and high EDS groups. Participants whose score fell on the median were included in the high EDS group (49% low EDS, 51% high EDS). The EDS was chosen as a measure of chronic stress due to its use in several recent pediatric BP association [16, 17, 56] and adult ET-1 association studies [29]. In addition, the relevance of item content was pertinent to our sample of adolescent AAs (e.g., and “in your day-to-day life, how often have you felt threatened or harassed or felt treated with less respect than other people”).

#### 2.2.5. Interventions

The 12-week intervention was conducted at two high schools during subjects' regular health education classes. Students taking these classes do not take physical education during that semester. Health education teachers implemented the training and were supervised by program instructors. Qualitative assessments of the teachers' program implementations were conducted weekly with three Likert scale items (0–4 scale), which assessed thoroughness, class attentiveness, and enthusiasm. Average instructors ratings across the 12-week intervention were 3.34 ± 0.26 for thoroughness, 3.28 ± 0.32 for class attentiveness, and 3.31 ± 0.27 for enthusiasm. There were no significant differences for instructor ratings across the treatment groups (all  *P*'s > .06).

### 2.3. Health Education Control (HEC)

Weekly health education lessons consisted of 50-minute sessions on CV health-related lifestyle behaviors based upon National Heart, Lung and Blood Institute guidelines for youth and included brochures, handouts, videotapes, discussions, and recommendations for increasing physical activity (e.g., walking, sports, etc.), establishing and maintaining prudent diet (e.g., reducing fat intake). HEC is a basic health education course and is considered a “usual practice” control group in this study.

#### 2.3.1. Life Skills Training (LST)

Weekly 50-minute sessions using selected components of the LST program involved group discussions, passive and active modeling, behavioral rehearsal, feedback, reinforcement, and behavioral homework assignments. The selected program components provided training in problem-solving skills, reflective listening, conflict resolution, and anger management to enhance social skills, assertiveness, and personal and social competence [59]. No relaxation or stress reduction techniques were given to the LST or HEC groups. 

#### 2.3.2. Breathing Awareness Meditation (BAM)

BAM is exercise one of the Mindfulness-Based Stress Reduction Program [60]. Practice involves focusing upon the moment, sustaining attention on the breathing process and passively observing thoughts. The individual sits upright in a comfortable position with eyes closed and focuses on diaphragm movements while breathing in a slow, deep, relaxed manner. Ten-minute sessions were conducted during health education class and at home each weekday. On weekends, subjects were instructed to practice 10-minute sessions twice daily. Self-reported BAM home practice adherence was 86.6 ± 7.4 percent. There were no significant differences between treatment groups on in-school attendance (*F*[2, 160] = 2.36, *P* = .10), HEC 81%, BAM 79%, and LST 88%.

### 2.4. Data Analysis

Change in values of daytime, nighttime, and 24-hour SBP, and DBP were compared using a series of 2 (ET- 1 genotype) by 3 (treatment group) by 2 (EDS group) analyses of variance of change scores (post- minus preintervention values) that covaried the respective preintervention values (ANCOVAs). ANOVA analyses were initially conducted on preintervention anthropometric and ambulatory BP values. In addition, changes in smoking (i.e., average cigarettes per week) and exercise (i.e., days/week engaged in sweat inducing physical activities) from the youth risk behavior surveillance system [61], and body mass index covarying preintervention values were examined among subgroups. There were no significant preintervention differences or pre- to post-changes found among the groups (all *P*'s > .10).

To further examine three-way interactions, two-way interactions and simple main effects across each level of a third variable were calculated using the same preintervention covariates. The third variable was chosen on the basis of the largest *F*-ratio from the two variables that only had two levels (i.e., ET-1 genotype or EDS group). Adjusted *F*-values (*F*_adj_) were calculated using the mean square for the analyses of interest divided by the mean square error term taken from the original model. All subsequent comparisons following the initial three-way ANCOVA were examined using Bonferroni adjusted alpha levels. 

 The series of analyses was originally completed with general linear modeling using EDS as a continuous variable which, as anticipated, revealed similar patterns of significant results and conclusions [62]. Given the complex interpretations of the multiple interactions that differentiate across groups, the previously described ANCOVA models using dichotomized median split EDS values are presented. 

## 3. Results

Preintervention anthropometric and ambulatory data are shown in Table 1. There were no significant differences between the treatment groups, ET-1 genotype, EDS, or treatment group by ET-1 genotype by EDS interactions on any of these parameters (all  *P*'s >.10).

### 3.1. Genotyping

Genotype frequencies included 100 participants homozygous for the G allele, 52 heterozygous G and T allele carriers, and 10 homozygous for the T allele. Frequencies were in Hardy-Weinberg equilibrium (*X* ^2^ = .87,  *df* = 1,  *P* = .35) [63]. Due to the small number of homozygous T allele carriers, participants classified either as heterozygous or homozygous for the T alleles were classified as “carriers” (38%), and homozygous G allele carriers were classified as “noncarriers” (62%).

### 3.2. Everyday Discrimination

A two-way (carrier status) x treatment group ANOVA was conducted on the EDS pretest scores and verified no significant baseline differences due to carrier status, treatment group, or the interaction between carrier status and genotype (all  *P*'s >.24). A *X* ^2^ analyses was used to examine the median split by treatment group dispersion rate and was not significant (*P* = .88). Finally, EDS change scores were examined to determine if any treatment group resulted in significant changes to EDS during the duration of the study. No significant changes in EDS scores by treatment group, ET-1 T allele carrier status, or their interactions were found (all  *P*'s > .29). Correlations between pre- and postintervention EDS scores were significant (*r* = .59,  *P* < .001) and indicate that these scores were stable throughout the study. 

### 3.3. Ambulatory Systolic Blood Pressure

#### 3.3.1. 24-Hour SBP

**Figure 2:**
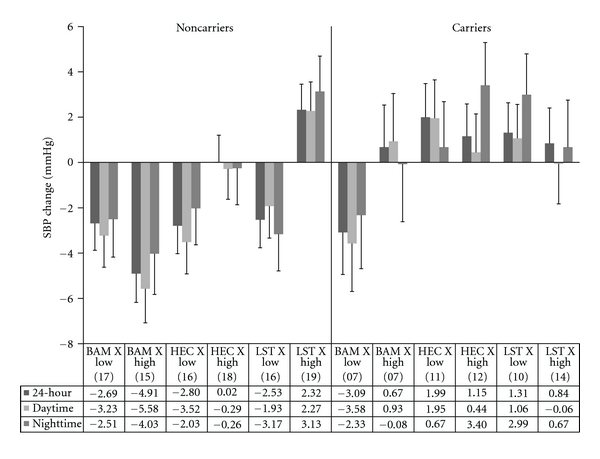
Change in ambulatory SBP as a function of everyday discrimination, ET-1 SNP carrier status, and treatment group. Note: BAM: breathing awareness meditation, HEC: health education control, LST: life skills training. Low: bottom 50th percentile for everyday discrimination; High: top 50th percentile for everyday discrimination. Values in parentheses indicate *n* for that subgroup.

The omnibus ANCOVA revealed significant main effects for ET-1 genotype (*F*[1, 149] = 7.57, *P* < .01) and treatment group (*F*[2, 136] = 4.73, *P* = .01) which were subsumed within an ET-1 genotype x treatment group x EDS group interaction (*F*[2, 149] = 4.14, *P* = .02). Results of the three-way interaction are depicted in Figure 2. Subsequent analyses examined the two-way interactions and simple effects for ET-1 carriers and noncarriers separately. No significant interactions or simple main effects for ET-1 carriers were found. Among ET-1 noncarriers, a significant simple main effect for treatment group (*F*_adj_[2, 149] = 4.46, *P* < .05) was subsumed within an EDS x treatment group interaction (*F*_adj_[2, 149] = 4.46, *P* < .05). Further simple effects analyses of treatment effects were separately conducted across the low and high EDS groups. There was no treatment effect among the ET-1 noncarriers who reported low EDS with groups showing comparable 24-hour SBP changes (range = −2.5 to −2.8 mmHg). There was a significant treatment group effect among those from high EDS backgrounds (*F*_adj_[2, 149] = 8.26, *P* < .05). Post hoc analyses revealed that those who received BAM showed greater decline than LST recipients (−4.9 versus +2.4 mmHg, *P* < .05).

#### 3.3.2. Daytime SBP

Significant main effects for ET-1 genotype (*F*[2, 146] = 5.38, *P* = .02) and treatment group (*F*[2, 146] = 3.90, *P* = .02) were subsumed within a three-way interaction involving the EDS group (*F*[2, 146] = 4.00, *P* = .02). The pattern of the three-way interaction was similar to that observed for 24-hour SBP (see Figure 2). Subsequent analyses revealed no significant interactions or simple main effects for ET-1 carriers. Among ET-1 noncarriers, a simple main effect for treatment group was found (*F*_adj_[2, 146] = 5.32,  *P* < .05). Post hoc examination revealed BAM participants showed greater reductions compared to LST (−4.4 versus +.19 mmHg, *P* < .05). Although not statistically significant, the subgroup of ET-1 SNP noncarriers who reported high EDS and received BAM exhibited the greatest reduction across all subgroups (−5.6 versus range of −3.5 to +2.3 mmHg). The only subgroup among ET-1 SNP carriers to show a reduction was those with low EDS that received BAM (−3.6 versus range of −.06 to +1.95 mmHg). 

#### 3.3.3. Nighttime SBP

A significant main effect for ET-1 genotype (*F*[1, 130] = 4.68, *P* = .03) and a trend for treatment group (*F*[2, 130] = 3.01, *P* = .06) were subsumed within a three-way interaction involving the EDS group (*F*[2, 130] = 3.01, *P* = .05). The pattern was similar to 24-hour and daytime SBP and is shown in Figure 2. Subsequent analyses revealed no significant interactions or simple main effects for the high EDS group. Among the low EDS group, a significant main effect for ET-1 carrier status was found (*F*_adj_[2, 130] = 4.13, *P* < .05; noncarriers = −1.7 versus carriers = +1.8 mmHg).

### 3.4. Ambulatory Diastolic Blood Pressure

#### 3.4.1. 24-Hour DBP

**Figure 3:**
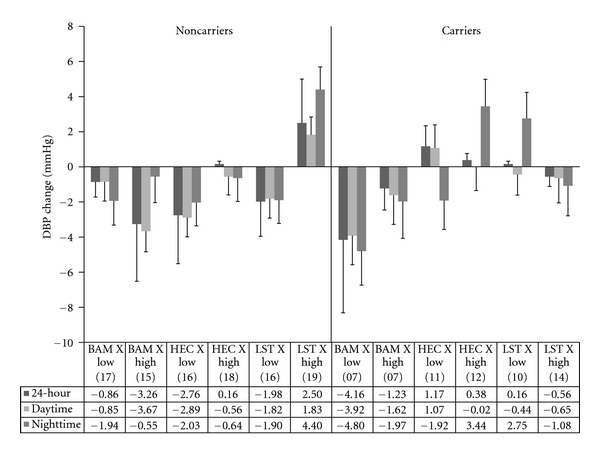
Change in ambulatory DBP as a function of everyday discrimination, ET-1 SNP carrier status, and treatment group. Note. BAM: breathing awareness meditation, HEC: health education control, LST: life skills training. Low: bottom 50th percentile for everyday discrimination; High: top 50th percentile for everyday discrimination. Values in parentheses indicate *n* for that subgroup.

A significant treatment group main effect (*F*[2, 149] = 4.58, *P* = .01) was subsumed within a significant three-way interaction (*F*[2, 149] = 5.38, *P* = .01).  Figure 3 displays the results of the three-way interaction. Subsequent analyses revealed no significant interactions or simple main effects for ET-1 carriers. Among ET-1 noncarriers a significant two-way interaction between EDS and treatment group (*F*_adj_[2, 149] = 6.56, *P* < .05) and a significant simple main effect among noncarriers (*F*_adj_[2, 149] = 4.28,  *P* < .05) were found. Further examination of treatment group effects among ET-1 noncarriers who reported low EDS was not significant and all treatment groups showed similar reductions in 24-hour DBP. Examination of treatment group among the ET-1 noncarriers who reported high EDS was significant (*F*_adj_[2, 149] = 8.38,  *P* < .05). Post hoc analyses revealed that participants who reported high EDS and received BAM were significantly different from those who received LST (−3.4 versus +2.5 mmHg, *P* < .05). The pattern of results is similar to the patterns found across SBP for ET-1 noncarriers; however, magnitude of change was less for DBP compared to SBP. For ET-1 carriers, more subgroups showed a reduction for DBP than SBP. However, those who received BAM displayed the best results and low EDS individuals showed better reduction than those high in EDS (−4.16 versus−1.23 mmHg). Interestingly, ET-1 carriers who received BAM and reported low EDS had the best improvement compared to other subgroups including noncarriers (−4.16 mmHg compared to range of −3.26 to +2.50 mmHg). 

#### 3.4.2. Daytime DBP

A significant treatment group effect (*F*[2, 146] = 3.26, *P* = .04) was subsumed within a three-way interaction (*F*[2, 146] = 3.52,  *P* = .03) which is displayed in Figure 3. Subsequent analyses showed no significant interactions or simple main effects for ET-1 carriers. Among ET-1 noncarriers, a significant two-way interaction between EDS and treatment group emerged (*F*_adj_[2, 146] = 4.77,  *P* < .05). Subsequent analyses showed no significant effects of treatment group among the low EDS subgroup. A treatment group effect was significant among the high EDS subgroup (*F*_adj_[2, 146] = 6.07,  *P* < .05) and post hoc analyses revealed that the BAM subgroup was significantly different from the LST subgroup (−3.6 versus +1.8 mmHg, *P* < .05). 

#### 3.4.3. Nighttime DBP

A significant treatment group effect (*F*[2, 130] = 3.91, *P* = .02) was subsumed within a two-way interaction involving ET-1 genotype and treatment group (*F*[2, 130] = 3.33, *P* = .04). When conducted separately, no significant interactions or simple main effects for ET-1 noncarriers were found. Among ET-1 carriers, there was a significant treatment group effect (*F*_adj_[2, 130] = 4.45, *P* < .05) and post hoc analyses revealed that participants who received BAM were significantly different from those who received LST (−3.6 versus +.74 mmHg, *P* < .05). Although a significant three-way interaction was not observed for nighttime DBP, for comparison purposes, the pattern of changes across ET-1 genotype, treatment group, and EDS group are displayed in Figure 3. 

## 4. Discussion

In this preliminary study, we hypothesized that individuals who were ET-1 SNP carriers would have greater difficulty in responding to any of our intervention treatments for improving ambulatory BP. In the following, we also expected ET-1 SNP carriers who reported high levels of discrimination to be the most difficult to show ambulatory BP reductions. Finally, we hypothesized that BAM would have greater beneficial impact upon ambulatory BP reduction compared to LST and HEC. Our hypotheses were partially supported. BAM participants exhibited greater reductions in 24-hour, daytime and nighttime SBP and DBP compared to the LST and HEC groups. The modulating influences of ET-1 SNP status and EDS were similar across all three ambulatory indices for SBP and DBP. Among ET-1 SNP carriers, the only subgroup to show a consistent reduction in SBP and DBP was BAM recipients who also reported low EDS. In many cases, the other subgroups showed relatively little reduction or even increases in BP. Among ET-1 SNP noncarriers, all three treatments were helpful in reducing BP among those who reported low EDS. Only BAM was beneficial in reducing BP among those who reported high EDS.

The ET-1 Lys198Asn SNP has been shown to play a significant role in vasoconstrictive mediated BP control in normotensive and hypertensive youth and adults [23, 24, 31, 34, 36]. Our findings provide further indirect support for the significant role of the ET-1 SNP in BP control among AAs. For all ambulatory SBP indices, ET-1 carrier status was a significant main effect showing fewer improvements compared to noncarriers. As noted above, BAM was the only treatment approach to have success in reducing SBP among ET-1 SNP carriers and only if they reported low EDS. It appears that among AAs, behavioral stress BP reduction programs such as BAM and LST may have difficulty in countering the combination of increased genetic propensity for stress-activated ANS imbalance/ET-1 activation and high frequency of environmental stress exposure [23, 24, 26, 34]. 

The cognitive skills-based program (LST) only benefitted ET-1 noncarriers and only if they reported low EDS. Participants who reported high EDS displayed a slight increase in ambulatory SBP. Acquisition of the LST skills (e.g., reflective listening, assertiveness without aggressiveness, etc.) may require the entire 12 weeks. Perhaps implementation of these newly learned skills in interpersonal conflict prone environments initially results in augmented vigilance and sympathetic/endothelial system activation, rather than reductions of such. The slight increase in ambulatory SBP among LST subjects who reported high EDS supports this rationale. Future studies would benefit from the utilization of repeated ambulatory BP and biomarker monitoring evaluations (e.g., total peripheral resistance, cardiac output, and nocturnal dipping), along with concomitant self monitoring of stressful encounters, affective states, coping responses, rumination, and using technological advances in cell phone capabilities.

Although provocative, these results should be interpreted cautiously. This was an exploratory ancillary analysis of an RCT, and subgroup cell sizes were relatively small. We examined potential confounding influences of sex, BMI, physical activity, and smoking and did not detect significant subgroup differences at preintervention or in response to the interventions. The issue of relatively small sample sizes within the three-way interactions can best be addressed by replication with larger sample sizes. One approach to consider would be to capitalize upon archival BP reduction RCTs that involved stress reduction programs and if not available, we would aquire DNA samples from the participants. We speculate that BP control improvements among BAM participants may have been partially a result of improved ANS balance/ET-1 activity. Several previous findings showed that BAM also reduced overnight sodium excretion purportedly through a reduction in sodium appetite. However, decreased sodium appetite is a correlate and not an adequate surrogate measure of ANS/ET-1 activity. The dynamic interplay between biological systems related to BP control warrants inclusion of biological measures of multiple systems and investigation of the interactions among pathways including the endothelial, ANS, renin-angiotensin and aldosterone, and HPA axis [25, 38].

 As noted earlier, retrospective post hoc analyses of meditation based BP RCTs involving prehypertensives and hypertensives (especially those involving AAs) may lend some support to whether the relationships found in this study translate to others. Finding similar patterns of ambulatory BP changes among ET-1 SNP status and other indices of chronic stress exposure would augment support for BAM as a viable approach for inclusion in nonpharmacologic programs aimed at the prevention of EH and CVD among certain subgroups of individuals (e.g., ET-1 SNP noncarriers, and carriers from low stress environments). The ease of BAM administration allows it to be practiced in virtual any setting (i.e., public schools, churches, recreation centers, and homes) adding to its utility to become part of multifaceted dissemination efforts to help decrease CVD morbidity and mortality [64].

 Unfortunately, our study found none of the behavioral stress reduction programs were beneficial among ET-1 SNP carriers who reported high EDS exposure. If our results are replicated, exploration of alternative behavioral and/or pharmacologic approaches that target endothelial function is warranted. Part of the study inclusion requirements was no current or planned engagement in a formal exercise, health promotion, or organized sports programs outside of regular school physical education courses, and the measures we used for physical activity were not differently influenced by the subgroups. However, behavioral interventions that are directed specifically at enhancing high-intensity physical activity may be beneficial. Aerobic exercise training has been shown to inhibit vasoconstrictive (e.g., endothelin-1) and promote vasodilatory (i.e., nitric oxide) mechanisms related to BP control providing evidence as a potentially effective therapeutic strategy [65–67]. Specific to the ET-1 LYS198ASN SNP, Rankinen et al. [67] found a two fold higher risk of hypertension among low aerobically fit carriers, whereas, aerobically fit carriers' hypertension risk was comparable to noncarriers. Additional research is needed to determine if physical activity can specifically benefit AA ET-1 carriers who report high levels of background stress. 

For some individuals, a gene x environment personalized behavioral intervention approach may not improve BP control to desired levels. If this occurs, pharmacogenomics-based primary prevention interventions should be considered. Several large-scale pharmacologic RCTs have proven beneficial in reducing onset of EH in prehypertensive adults [68, 69]. Among ET-1 SNP carriers, an endothelin type A receptor antagonist may help foster vasodilation-mediated BP control. In a recent study, Weber et al. found Darusentan, a selective endothelin type A antagonist, to control treatment resistant hypertension [70]. 

## 5. Conclusions

In summary, the findings provide preliminary evidence of some of the underlying contributors that may have moderated BP reductions but were not examined in previous meditation RCTs. “One-size fits all” approaches to primary and secondary preventive health-care are being replaced with strategies described as preventive, predictive, personalized, and participatory [71]. Increasingly, behavioral and pharmacologic interventions are being tailored on the basis of individual's underlying genetic propensities and environmental factors (e.g., attitudes, stress exposure, etc.). Personalized medicine is in its infancy, but eventually, via empirical scrutiny, more efficacious best practice prevention and treatment approaches will evolve. The end result will help reduce the incidence of chronic diseases and improve the quality and longevity of life among those with these diseases.

## Figures and Tables

**Table 1:** Baseline anthropometric characteristics.

Characteristic	BAM (*n* = 46)	LST (*n* = 59)	HEC (*n* = 57)
Age (years)	15.0 ± 0.6	15.0 ± 0.7	15.2 ± 0.8
Sex (male/female)	16/30	27/32	25/32
Weight (kg)	66.3 ± 15.8	70.8 ± 17.3	66.8 ± 16.5
Height (cm)	163.4 ± 8.3	167.5 ± 8.6	163.6 ± 7.9
BMI (kg/m^2^)	24.8 ± 5.3	25.1 ± 5.0	24.9 ± 5.9
LYS198ASN (TT/TG∣GG)	14∣32	25∣34	23∣34
EDS (high/low)	22/24	29/30	30/27
24-hour SBP	119.3 ± 6.1	119.8 ± 6.5	121.8 ± 6.8
Daytime SBP	124.0 ± 6.4	123.7 ± 6.5	126.2 ± 7.5
Nighttime SBP	109.1 ± 6.6	110.6 ± 8.7	111.16 ± 8.1
24-hour DBP	68.6 ± 5.6	68.0 ± 5.5	69.3 ± 6.2
Daytime DBP	73.4 ± 5.9	72.5 ± 5.5	73.9 ± 6.6
Nighttime DBP	57.9 ± 6.1	57.9 ± 6.7	58.7 ± 5.7

## Abbreviations

AA: African American
ABP: Ambulatory blood pressure
BAM: Breathing awareness meditation
BP: Blood pressure
CVD: Cardiovascular disease
DBP: Diastolic blood pressure
EDS: Everyday discrimination scale
EH: Essential hypertension
ET-1: Endothelin one
HEC: Health education control
LST: Life skills training
PCR: Polymerase chain reaction
SBP: Systolic blood pressure
SNP: Single nucleotide polymorphism
ANS: Autonomic nervous system.
